# Prediction model for drug response of acute myeloid leukemia patients

**DOI:** 10.1038/s41698-023-00374-z

**Published:** 2023-03-24

**Authors:** Quang Thinh Trac, Yudi Pawitan, Tian Mou, Tom Erkers, Päivi Östling, Anna Bohlin, Albin Österroos, Mattias Vesterlund, Rozbeh Jafari, Ioannis Siavelis, Helena Bäckvall, Santeri Kiviluoto, Lukas M. Orre, Mattias Rantalainen, Janne Lehtiö, Sören Lehmann, Olli Kallioniemi, Trung Nghia Vu

**Affiliations:** 1 Department of Medical Epidemiology and Biostatistics, Karolinska Institutet, Stockholm, Sweden; 2 School of Biomedical Engineering, Shenzhen University, Shenzhen, China; 3 Department of Oncology Pathology, Karolinska Institutet, Science for Life Laboratory, Stockholm, Sweden; 4 Institute for Molecular Medicine Finland, University of Helsinki, Helsinki, Finland; 5 Department of Medicine Huddinge, Karolinska Institutet, Unit for Hematology, Karolinska University Hospital Huddinge, Stockholm, Sweden; 6 Department of Medical Sciences, Hematology, Uppsala University Hospital, Uppsala, Sweden

**Keywords:** Computational biology and bioinformatics, Cancer

## Abstract

Despite some encouraging successes, predicting the therapy response of acute myeloid leukemia (AML) patients remains highly challenging due to tumor heterogeneity. Here we aim to develop and validate MDREAM, a robust ensemble-based prediction model for drug response in AML based on an integration of omics data, including mutations and gene expression, and large-scale drug testing. Briefly, MDREAM is first trained in the BeatAML cohort (*n* = 278), and then validated in the BeatAML (*n* = 183) and two external cohorts, including a Swedish AML cohort (*n* = 45) and a relapsed/refractory acute leukemia cohort (*n* = 12). The final prediction is based on 122 ensemble models, each corresponding to a drug. A confidence score metric is used to convey the uncertainty of predictions; among predictions with a confidence score >0.75, the validated proportion of good responders is 77%. The Spearman correlations between the predicted and the observed drug response are 0.68 (95% CI: [0.64, 0.68]) in the BeatAML validation set, –0.49 (95% CI: [–0.53, –0.44]) in the Swedish cohort and 0.59 (95% CI: [0.51, 0.67]) in the relapsed/refractory cohort. A web-based implementation of MDREAM is publicly available at https://www.meb.ki.se/shiny/truvu/MDREAM/.

## Introduction

Acute myeloid leukemia (AML) is an aggressive blood cancer with high genetic heterogeneity and varying responses to therapy. Traditionally, targeted cancer therapies, including AML, are designed based on genetic biomarkers. In the classification of the World Health Organization (WHO) in 2008^1^, genomic features such as fusion genes, including PML-RARA, RUNX1-RUNX1T1, DEK-NUP214, CBFB-MYH11, and mutations of CEBPA and NPM1, determined molecular subtypes in adult AML. A more recent study^2^ proposed 11 molecular subtypes, each associated with specific diagnostic and clinical features. The first targeted therapy of AML used all-trans retinoic acid (ATRA) targeting to PML-RARA fusion of Acute Promyelocytic Leukemia (APL), a subtype of AML characterized by the translocation of the retinoic acid receptor *α* (RARA) on chromosome 17 and promyelocytic leukemia (PML) gene on chromosome 15^3^. Recently, Midostaurin, a fms-like tyrosine kinase 3 (FLT3) inhibitor, was approved for use in AML with FLT3 mutations in 2017^4^.

Despite some encouraging successes, predicting therapy responses of AML patients remains highly challenging^5^. The patterns of the mutations and genetic abnormalities within and across patients are highly complex, and only a few targeted therapies have been developed for AML. The genetic complexity can also reduce the efficacy of a given therapy or make the patient stratification inaccurate. For example, >40% of AML patients with FLT3+ fail to respond to Midostaurin, but >30% of FLT3– cases potentially respond to the drug^6^. In clinical practice, because of the complexity of AML and its potential treatments, the selection of therapy is often subjective, decided based on the experience or intuition of the clinician^7^. These suggest there is still a need for an effective computational prediction model for the drug response of individual AML patients.

To date, there are a few studies on the methodologies for drug-response prediction using omics data for AML. In 2016, Ammad-ud-din and colleagues developed a kernelized Bayesian matrix factorization method^8^, but it was validated in a small set of AML cell lines (*n* = 6). Similarly, Gerdes et al.^9^ introduced a machine-learning approach to predict and rank anti-cancer drugs on 26 AML cell lines using proteomics data. Lee et al. presented the MERGE score^10^ to identify gene expression markers and predict drug sensitivity of AML cases. However, it was also trained and validated in a limited number of AML cases (*n* = 34 AML patients and *n* = 14 AML cell lines), which cannot capture the wide AML heterogeneities.

In this study, we develop MDREAM (Monotherapy Drug Response prediction for AML) using integrated omics data. MDREAM is trained and first validated using the BeatAML cohort (*n* = 278 for training and 183 for validation)^11^. The prediction is based on 122 ensemble models, each corresponding to a drug. The results show MDREAM is well validated in the BeatAML validation set and in the external validation set using an in-house cohort (Clinseq) including 45 Swedish AML patients, and also a public acute leukemia dataset (LeeAML) (*n* = 12)^10^. For the prediction of each patient, we introduce a confidence score to measure the prediction uncertainty of individual drugs, guiding the practical use of the prediction for the patient. MDREAM is implemented in a publicly available web application at https://www.meb.ki.se/shiny/truvu/MDREAM/.

## Results

### AML-specific drug response prediction model

**Fig. 1:**
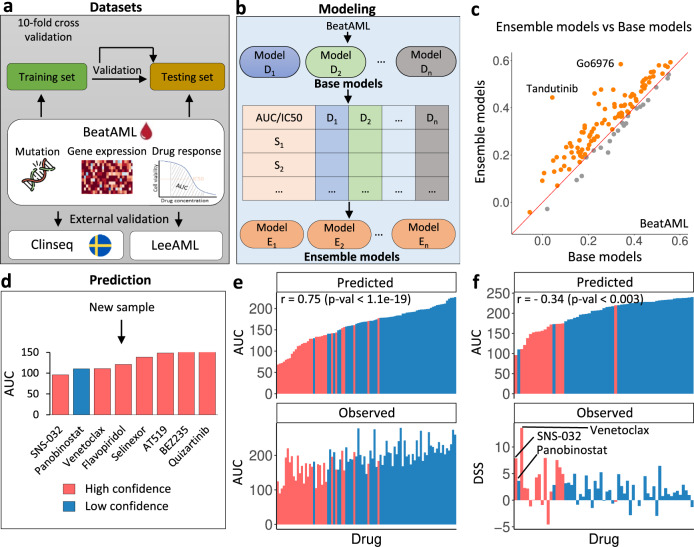
Overview of MDREAM. **a** Omics data of the BeatAML cohort are used to train and validate the performance of the model. We further use the Clinseq cohort and the LeeAML cohort as two external validation datasets. **b** The ensemble-based prediction model uses the stacking approach including two layers: (1) the first layer contains 122 base models for prediction (*D*_1_, *D*_2_,..., *D*_*n*_), each is built from the data of a single drug; (2) the second layer consists of 122 ensemble models (*E*_1_, *E*_2_,..., *E*_*n*_) corresponding to 122 drugs. Each ensemble model is built based on the prediction output of the first layer. An advantage of ensemble learning is that it can exploit the correlation of drug sensitivity between drugs. **c** Comparison of performance between the base models (x-axis) and the ensemble models (y-axis) in the BeatAML validation set using the correlation between the predicted and observed response of individual drugs. Orange points above the diagonal line indicate higher correlation—better performance—achieved by the ensemble models. **d** Prediction results with information on confidence score for a new patient sample from the external validation dataset. This plot shows the results of the patient from the ClinSeq cohort with a median Pearson correlation between predicted AUC and observed drug sensitivity. The drugs with low AUC and high confidence (confidence score >0.75) might be of interest for further investigation in the treatment of that patient. **e**, **f** Waterfall plots of the predicted AUC and observed drug sensitivities of the patient with median Pearson correlation from the BeatAML validation set (**e**) and the Clinseq dataset (**f**). Note that the negative correlation in **f** panel is as expected since the AUC and drug sensitivity score (DSS) have opposite directions: lower AUC and higher DSS correspond to better drug response.

Figure 1 describes the overview of MDREAM. Briefly, MDREAM takes input omics data, including gene expression and mutation profiles, and drug sensitivity data from the BeatAML cohort for training the drug-response prediction model, then the model is validated using both internal and external datasets, Fig. 1a. A total of 461 AML patients and their ex-vivo drug sensitivities from 122 small molecule inhibitor from the BeatAML cohort are included in the analysis. Two prediction models are built separately for two available metrics of drug sensitivity in the cohort: IC_50_ and area under the curve (AUC), where a lower IC_50_/AUC indicates a better drug response.

The model of MDREAM is built using the stacking approach, Fig. 1b, using relevant biological features extracted from the omics data; see further details in “Prediction model and genomic features for drug response of AML”. The ensemble models of the stacking approach are generally more advanced than the base models because they can utilize the aggregation of base models to improve the robustness of the prediction. The ensemble approach also fits the problem of drug response prediction in this study. Indeed, it is common that a drug is not screened across all patients, which can limit the performance of the base model. Furthermore, many drugs sharing similar gene targets can have highly correlated responses, which can also be efficiently utilized by the ensemble models. Figure 1c shows the comparison of prediction performance between the ensemble models and the base models in the BeatAML validation set. Each point presents the correlation between the predicted and observed response for each single drug. Orange points above the diagonal line indicate drugs with higher correlation (97/122 drugs, 80%), indicating the ensemble models to be better than the base models. The similar comparison in Clinseq also shows the higher performance of the ensemble models for the majority of drugs (50/76 drugs, 66%) compared to the base models (see Supplementary Fig. 1).

For a new patient sample, MDREAM reports the predicted IC_50_/AUC of 122 drugs and also provides the confidence score (CS) for each prediction, Fig. 1d. The value of CS reflects the consistency of the prediction across bootstrap replicates of the training data, see more details in the section “Confidence score for drug-response prediction”. Figure 1e shows the prediction performance of the model for the patient from the BeatAML testing set with a median correlation between prediction and observation (*r* = 0.75, *p* value <1.1e-19). Each drug is presented by a bar and the color of the point represents its confidence score. The y-axis of the two panels are ranked by the drug response prediction. Drugs predicted to have a good response, and with a high confidence score appear consistently on the left side of the panels, e.g., elesclomol, quizatinib, and foretinib.

Similarly, Fig. 1f shows the individual with a median correlation from the Clinseq cohort (*r* = –0.34, *p* value < 0.003). The drug sensitivity in the Clinseq cohort is measured using drug sensitivity score (DSS)^12^, where a higher DSS indicates a better drug response, so we expect a negative correlation between the predicted AUC and the observed DSS. We note that the data used in Fig. 1d and f are from the same patient. Among the top three predicted drugs, sns-032 and venetoclax achieved the lowest predicted response at AUC = 96.0 (95% CI: [94.2, 125.2]) and 110.9 (95% CI: [90.9, 129.7]) with high confidence scores of 1.0 and 0.99 respectively, so might be worth further investigation for the patient. Ponobiostat shows a non-confident prediction with of low confidence score, indicating it is unlikely that the patient would be a good responder to the drug as predicted; indeed, the observed DSS for the patient was low.

The AML-context-driven features used for the drug-response prediction are described in the section “Prediction model and genomic features for drug response of AML”. To explore the contribution of these features to the prediction, we apply the variable importance method of Fisher et al.^13^ for the SVM base model of every drug. The method produces a score for each feature, presenting the contribution of that feature to the prediction performance of the drug. The top ten most important features of each drug are provided in Supplementary Data 1. Taking Venetoclax as an example, the method reports TSPAN10, NFIA, AARD, DACH1, AGR2, AATK-AS1, BCL2, DHRS2, LRP1, and MYO7A as the top important features for the prediction. Among those, BCL2 is the primary target of that drug. However, further investigation of the other important features of Venetoclax and other drugs is beyond the scope of this study.

### Validation of MDREAM in the BeatAML cohort

**Fig. 2:**
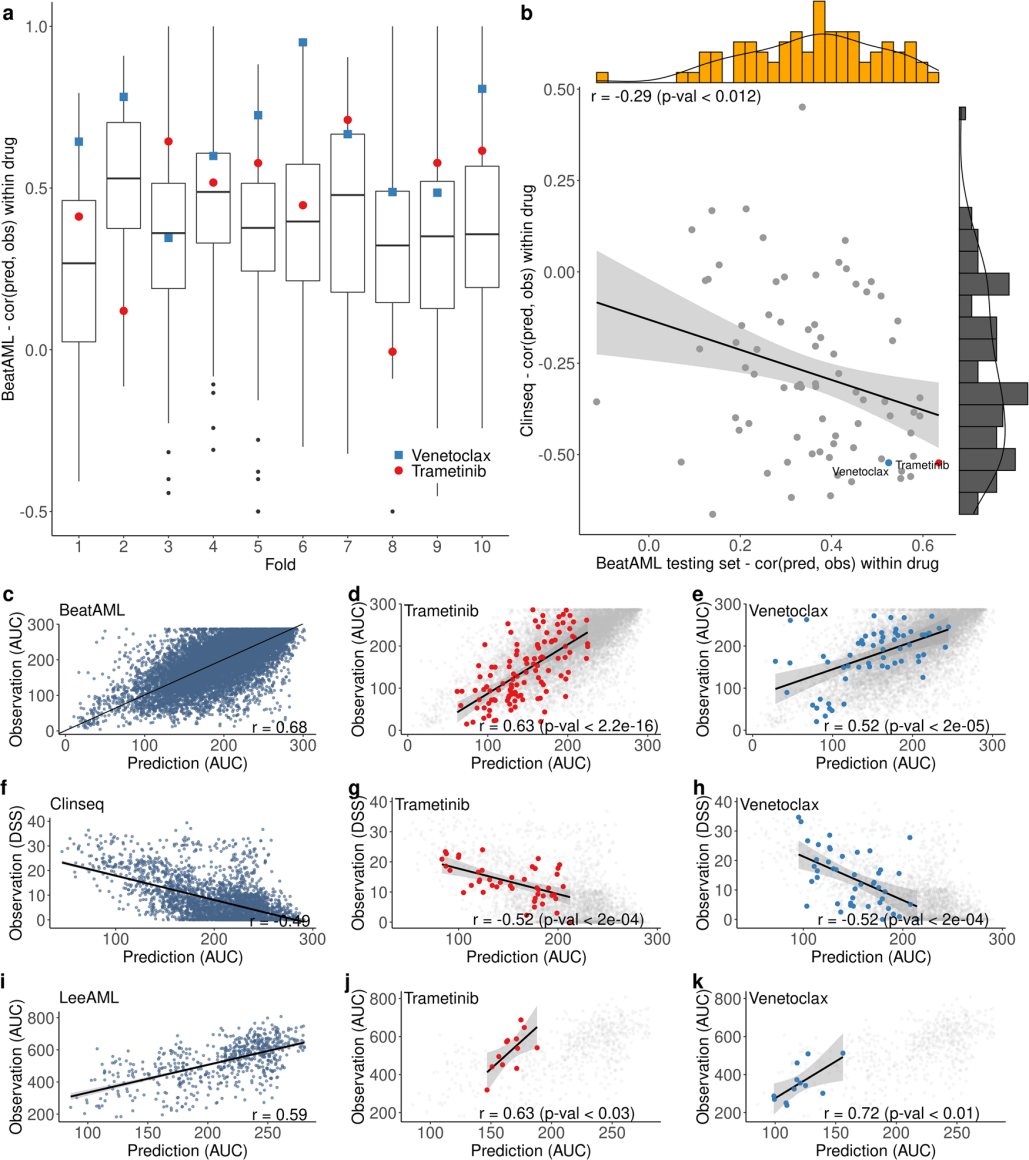
Performance of MDREAM in BeatAML, Clinseq, and LeeAML. **a** Boxplots show the correlation of individual drugs from the tenfold cross-validation in the training set. Each boxplot displays the interquartile range (IQR) between the 25th percentile (the lower boundary) and the 75th percentile (the upper boundary). The center line of the box presents the median, and the whiskers are within the 1.5 IQR value. The points (blue or red) in each boxplot represent the correlation results of venetoclax and trametinib. **b** Prediction performances of the BeatAML testing set (x-axis) versus the Clinseq dataset (y-axis) across 76 overlapping drugs using the correlation between predicted values and observed values. The drugs at the bottom right indicate the drugs with good predictions in both cohorts. **c**–**e** Predicted AUCs vs observed AUCs of all data points (**c**) and individual drugs, including Trametinib (**d**) and Venetoclax (**e**) from the BeatAML testing set. **f**–**k** Similar scatter plots for predicted AUCs vs observed DSSs of the Clinseq dataset and LeeAML dataset, respectively.

We first evaluate the performance of MDREAM using an independent testing set and 10-fold cross-validation of the training set; see Fig. 1a. The BeatAML patients are split based on their center IDs into the training set (60%, *n* = 278) and an independent testing set (40%, *n* = 183). The boxplots in Fig. 2a summarize the cross-validation results in the training set. The x-axis indicates the index of each of the ten folds of the cross-validation. The y-axis presents the correlation between predictions and observations of individual drugs. The median correlation of each fold ranges from 0.26 to 0.52, and the median correlation across tenfolds is 0.36.

To predict the BeatAML testing set, we use the whole training set to build the prediction model. For this testing set, the median correlation between the predicted and observed values for individual drugs is 0.35, which is close to the above cross-validation result, indicating no over-fitting issue in the training model. The correlation data of 76 drugs overlapping with the Clinseq cohort are shown in Fig. 2b and also reported in Supplementary Data 2. In this figure, each point presents a single drug with the corresponding correlation value in the x-axis. The histogram on top of the figure shows the correlation distribution of these drugs. As the results, among 76 overlapped drugs, trametinib achieves the highest correlation (*r* = 0.63), while venetoclax also reports good prediction (*r* = 0.52).

Figure 2c shows a high overall correlation between the predicted AUC and the observed AUC of the testing set (*r* = 0.68, 95% CI: [0.64, 0.68]). This plot includes all data points of the testing set, each point represents a (patient, drug)-pair. The patient with the median correlation (*r* = 0.75) between the predicted and observed values across drugs is depicted in Fig. 1e, while the distribution of the correlation for all individual patients is provided in Supplementary Fig. 2. We further compare MDREAM with a simple model (drug-average model) which uses the average of responses of each drug from the training data. Thus, for a single drug, the drug-average model uses the average value for all patients. The correlation between the predicted values from the drug-average model and the observed values is calculated for each patient in the validated set. As a result, see Supplementary Fig. 2, MDREAM achieves better performance than the drug-average model for most patients (62%, orange points below the diagonal line). The prediction results of trametinib and venetoclax are highlighted in Fig. 2d, e.

For the prediction of IC_50_, we obtained slightly lower median correlations of individual drugs, *r* = 0.35 and *r* = 0.33 for the cross-validation and the testing set, respectively. This can be due to a drawback in the IC_50_ data that a majority (>40%) have a constant upper boundary (IC_50_ = 10). Further details of the results for IC_50_ are provided in Supplementary Data 2.

### Validation of MDREAM in the Clinseq cohort

We include all samples of the BeatAML cohort (*n* = 461) to build MDREAM and use the Clinseq cohort for validation. The Clinseq cohort is based on 45 Swedish AML patients and 76 drugs overlapping with 122 drugs in the BeatAML cohort. There are a total of *n* = 3420 drug response data, which are measured using Drug Sensitivity Score (DSS)^12^. The summary of the correlation for individual drugs is presented by the histogram at the right-most side of Fig. 2b. The median correlation across all individual drugs is −0.32, slightly less than the results of the BeatAML cohort (*r* = 0.35). We bootstrap the BeatAML data (*n* = 100 times), then apply the same method for model building and prediction to get the bootstrap correlation values, and finally obtain a 95% confidence interval of –0.33 to –0.25 for the correlation (Supplementary Fig. 3).

Figure 2f presents a well validation of MDREAM for the Clinseq cohort with the overall Spearman correlation of –0.49 (95% CI: [–0.53, –0.44]) between the predicted AUC and the observed DSS across all data points. The patient with the median correlation (*r* = –0.34) between the predicted and observed values across drugs is depicted in Fig. 1f, while the distribution of the correlation for all individual patients is provided in Supplementary Fig. 2. MDREAM also obtains better performance in 71% patients than the drug-average model, see Supplementary Fig. 2.

Figure 2b also shows a good concordance of the prediction results of 76 drugs between the two cohorts (*r* = –0.29). In addition, the two drugs Trametinib and Venetoclax that have good results in the BeatAML cohort also show good performance in the Clinseq cohort, Fig. 2g, h. It is worth noting that the drug response metrics are different and independent between the two cohorts, suggesting the robustness of MDREAM. For the prediction based on IC_50_, the correlation of all data points (*r* = –0.51) and the median correlation of individual drugs (*r* = –0.29) are comparable to the AUC-based results (See Supplementary Data 2).

### Validation of MDREAM in the LeeAML cohort

We apply a similar procedure to validate MDREAM in the LeeAML dataset^10^. The dataset contains genomic data from 12 relapsed/refractory acute leukemia patients and 624 drug response profiles from 52 drugs that overlap with the 122 drugs in the BeatAML dataset. Similar to the validation in the BeatAML cohort (Fig. 2c) and the Clinseq cohort (Fig. 2f). Fig. 2i illustrates a high overall correlation between the predicted AUC and observed AUC of 624 (drug, patient) points from the LeeAML dataset (*r* = 0.59, 95% CI: [0.51, 0.67]). The distribution of the correlation over all drugs for all individual patients (the median of 0.63) is provided in Supplementary Fig. 2. In comparison with the drug-average model, MDREAM gains better correlation for all patients, see Supplementary Fig. 2. The correlations for all individual drugs are also shown in Supplementary Fig. 4. Trametinib and venetoclax, which have good performances in the BeatAML cohort, also archive top results in the LeeAML cohort, Fig. 2j, k. A negative correlation of some drugs in the LeeAML cohort might be due to the small number of samples (*n* = 12).

### Uncertainty assessment using a confidence score

**Fig. 3:**
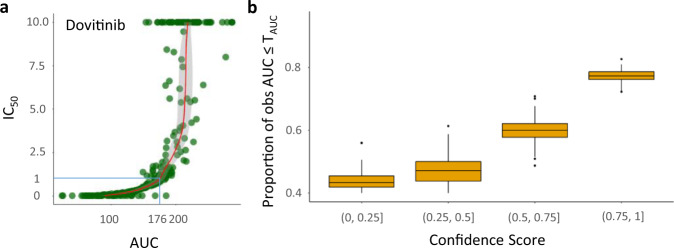
Confidence score for drug response prediction. **a** Relationship between AUC and IC_50_ for the drug dovitinib in the BeatAML dataset; this is used to determine the threshold of good response based on the AUC (*T*_AUC_). **b**. The proportion of validated good responders (observed ${{{\rm{AUC}}}}\le {T}_{{\mathrm{AUC}}}^{D}$ in the BeatAML testing set) increases with higher confidence scores. Each boxplot displays the interquartile range (IQR) between the 25th percentile (the lower boundary) and the 75th percentile (the upper boundary). The center line of the box presents the median, and the whiskers are within the 1.5 IQR value. ${T}_{{\mathrm{AUC}}}^{D}$ is the threshold of AUC for good drug responses in drug D. The x-axis represents four categories of confidence scores.

Given data from a new patient, theoretically, the prediction model can report the predicted responses of 122 drugs; those predicted to have good responses may be considered further. But, how confident are we in the prediction? In the BeatAML cohort, IC_50_ ranges from 0 to 10, where lower IC_50_ indicates better drug response. So we first define “good response” in terms of IC_50_ ≤ 1 and translate the threshold for the AUC (to get *T*_AUC_) based on the drug-specific relationship between IC_50_ and AUC; see Fig. 3a. The confidence score (CS) of each prediction is then computed using the bootstrap samples; see section “Confidence score for drug-response prediction”. The confidence score ranges from 0 to 1, and a higher confidence score indicates higher consistency in obtaining a good-response prediction across the bootstrap replications of the data.

We expect predictions with higher confidence scores to have a higher level of validation. Indeed, Fig. 3b shows the proportion of good-response predictions that are validated in the BeatAML testing set increases with the confidence score. In this plot, the x-axis presents four increasing categories of confidence scores, and the y-axis shows the proportion of validated good-response. From the plot, the median of validated good-response proportions among predictions with a confidence score of less than 0.50 is less than 50%. If we keep only predictions with a confidence score >0.75, the validated proportion reaches around 77%. We found a similar trend for the results based on IC_50_, Supplementary Fig. 5.

The example in Fig. 1c shows an application of the confidence score to identify drugs with low and high confidence of an individual in the Clinseq cohort. Among top three predicted good-response drugs, only sns-032 and venetoclax show high confidence (score >0.75), which might be considered further individualized therapy. Figure 1e shows the prediction results of all drugs and their confidence scores of the same patient in Fig. 1c. A similar plot for a patient of the BeatAML testing set is found in Fig. 1d.

To investigate the advantage of the confidence score (CS) over the approach using the most extreme predictions, we compare the results of K drugs with a high confidence score (CS > 0.75) with top K extreme-prediction drugs ranked by the predicted AUCs for each patient in the BeatAML testing set. We summarize the correlations between the predicted and observed AUCs for each group of all patients for comparison in Supplementary Fig. 6. The plot shows that the group of drugs selected by the confidence score (the orange boxplot) obtains a higher correlation than the group of drugs from the most extreme prediction (the green boxplot). Thus, ranking drugs by the confidence score is better than ranking by the most extreme predictions.

### Drug response in relation to AML subtypes

AML shows highly heterogeneous clinical phenotypes and molecular characteristics, which greatly complicate its clinical management. In a recent work^2^, Papaemmanuil and colleagues propose a classification of 11 distinct AML molecular subtypes, suggesting molecular driving mechanisms of the disease. Many molecular subtypes are concordant with known clinical subtypes, such as those associated with PML-RARA, RUNX1-RUNX1T1, or CBFB-MYH11 fusions, etc. Characterizing the relationship between these molecular subtypes and the drugs might inform us of the molecular mechanism of a drug in a responsive patient.

We investigate the association between the molecular subtypes and drugs by assessing the functional interaction between drug-target genes and the genes that are specific to the subtypes. The molecular subtype-specific genes are collected from the recent work of Mou et al.^14^, and the target genes of each drugs are collected from DrugBank database^15^. For each pair of (subtype, drug), we apply the network enrichment analysis (NEA)^16^ to obtain the enrichment score (*z*-score) between the two genesets; see further details in the section “AML molecular subtypes in relation to drug response”. A higher *z*-score (>1.96) indicates a stronger functional interaction between the target genes of the drug and the subtype-specific genes of the subtype.

**Fig. 4:**
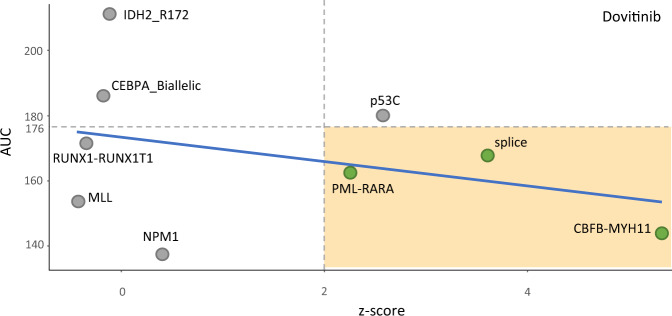
AUC versus *z*-score for different AML molecular subtypes. Each point represents samples of an individual subtype group. The y-axis is the median value of drug response (AUC) of each subtype group. The *z*-score (x-axis) is a statistic of network enrichment analysis (NEA), which is used to assess the interaction between the subtype-specific genes and the drug-target genes in the functional gene network. A higher *z*-score indicates a stronger interaction than expected in a random permutation of the network. The blue line is the linear regression line. The yellow rectangle suggests some interesting subtypes, which respond to the drug potentially via the functional interaction between the subtype-specific and the drug-target genes.

For a drug, the enrichment scores can vary across subtypes and correlate with the sensitivity of the drug. Figure 4 shows such an example from the drug dovitinib of the BeatAML cohort. In this plot, each point represents an AML subtype, the x-axis presents the *z*-score, and the y-axis expresses the median AUC of patients in each subtype. The Spearman correlation between AUC and *z*-score is –0.23. The subtypes with a *z*-score greater than 1.96 (the vertical dash line), including PML-RARA, splice, CBFB-MYH11, and p53C are significant (*p* value < 0.05). Among those, dovitinib has a good response to the first three subtypes under the horizontal dash line. The plot indicates that dovitinib has a good response for the patients in PML-RARA, splice and CBFB-MYH11 subtypes, and the good response could potentially be explained by the functional interaction between its target genes and subtype-specific genes of the subtypes. Note that dovitinib also shows a good response for RUNX1-RUNX1T1, MLL, and NPM1 subtypes; however, the plot tells us that we cannot explain why the drug works for those subtypes based on the interaction between the target genes and the subtype-specific genes. This subtype-drug interaction analysis is integrated into MDREAM; a classifier to predict the subtype of a new patient is also provided; see section “Prediction model and genomic features for drug response of AML”.

### MDREAM in comparison with existing methods

An early version of MDREAM was developed during our participation in the CTD-squared BeatAML DREAM challenge^17^. The DREAM challenge is a rigorous challenge for a competition of 28 international research groups working intensively for a long time (6 months) with extensive validation to evaluate competing methods. The challenge had two sub-challenges: (i) monotherapy drug response prediction and (ii) clinical response prediction. MDREAM ranked third in the drug response prediction sub-challenge, though statistically, there was little difference between the top four teams; see the final leaderboard of the DREAM challenge (our team was called CBA)^17^. It is noted that both MDREAM in this study and the competing methods in the challenge use the same data provided from the BeatAML article^11^.

Ammad-ud-din et al. applied Kernelized Bayesian Matrix Factorization (KBMF) model for prediction and computed the overall correlation between the predicted and the observed response measured from wet-lab validation of 48 drug-response profiles from eight drugs and six AML cell lines^8^. Their study reported a validated overall correlation of 0.44, compared to our results of 0.68, –0.49, and 0.59 for the validation of MDREAM in the BeatAML, Clinseq, and LeeAML cohorts, respectively. When applying the KBMF model to train and test on the same datasets used for MDREAM in this study, the median correlations across individual drugs show that MDREAM also outperforms the KBMF method in both BeatAML testing set (*r* = 0.35 vs 0.24) and Clinseq dataset (*r* = −0.32 vs −0.05), see Supplementary Fig. 7. We exclude the LeeAML dataset from the analysis due to its small sample size (*n* = 12). In the BeatAML testing set, 106/122 drugs (87%, orange points) have a better performance in MDREAM. Similarly, MDREAM also has the majority of the drugs (80%) with better correlation than KBMF does.

We further compare the features used by MDREAM with the clinically relevant gene biomarkers of 7 AML cell types from Zeng et al.^18^. Particularly, we collect the top five genes specific to each cell type which are provided in the article. Next, we replace the current data features of MDREAM with the gene expression of these gene biomarkers to build a cell type-markers-based model for comparison with MDREAM. Thus, the cell type-markers-based model and MDREAM use exactly the same prediction framework, so their results can be used for comparison of the importance of features used in the models. Supplementary Figure 8a compares the performance of MDREAM vs the cell type-markers-based model using the BeatAML testing set. Each point represents the Spearman correlation between the predicted AUC and the observed AUC values of an individual drug (a higher positive correlation is better). A large proportion of points (102 of 122, 84%) are below the diagonal line, indicating that MDREAM has better performance than the cell type-markers-based model in most drugs. A similar result is also found for the Clinseq dataset (see Supplementary Fig. 8b). In this plot, each point indicates the Spearman correlation between predicted (AUC) and observed (DSS) values of an individual drug where a lower negative correlation indicates better response. In 63 of 76 drugs (83%), MDREAM shows a better performance. We further investigate whether these cell-type gene biomarkers bring added value to the performance of MDREAM. To do this, we combine the features of both the cell type-markers-based model and MDREAM to build a combined model (MDREAM + cell type-markers model). The high correlations for both datasets in Supplementary Fig. 9 indicate that adding the cell-type gene biomarkers does not significantly impact the MDREAM performance.

## Discussion

We have developed and validated MDREAM, a computational method for drug response prediction in AML based on integrating omics data, including mutations and gene expression, and large-scale drug response assay data. We also proposed a confidence score to convey the uncertainty of the predictions. MDREAM is trained using the BeatAML cohort, and validated in the BeatAML cohort and two external cohorts. We further investigate AML subtype genes in association with drug sensitivity.

The establishment of pharmacogenomic datasets (e.g., NCI-60 database^19^; Genomics of Drug Sensitivity in Cancer (GDSC) project^20^; Cancer Cell Line Encyclopedia (CCLE) project^21^) enables the development of drug response prediction methods in pan-cancer. Most of these pan-cancer methods train predictors by using the gene expression profiles of cancer cell lines, which has been shown as the most informative source for drug response prediction^22,23^. However, these pan-cancer drug response predictors are usually not optimal for specific cancer, since each cancer has a limited number of cell lines which cannot comprehensively capture the heterogeneity and diversity of cancer. Thus, new efforts have been focused on developing cancer-specific drug response predictions for several cancers, including breast cancer^24,25^ and colorectal cancer^26,27^. AML is also targeted under the effort of developing drug response prediction methods and applications of few studies^8–10^.

MDREAM would provide useful information supplement to simple clinical guidelines. For example, we attempt to explore the advantage of MDREAM over Midostaurin for patients with FLT3 mutation. We select 29 patients carrying FLT3 mutation from the BeatAML testing set for the evaluation. The observed drug responses of these patients using Midostaurin are compared to those using Top-1 drug and Top-5 drugs with the highest confidence scores discovered by MDREAM. The results in Supplementary Fig. 10 show that the observed AUCs of both Top-1 drug and Top-5 drugs are significantly lower than those of Midostaurin, indicating that MDREAM can suggest drugs with a better response than Midostaurin. Since the genetic complexity of AML leads to >40% of AML patients with FLT3 mutation fail to respond to Midostaurin^6^, the suggested drugs from MDREAM might provide useful information for clinical application.

As its main strength, MDREAM had been previously compared and seen to perform well against a large number of competing models. It is built using a large cohort of AML patients, and validated in three distinct cohorts. The measurements of drug sensitivities in the BeatAML and the Clinseq cohorts are different: BeatAML cohort reports AUC and IC_50_ for drug response, while Clinseq uses the drug sensitivity score (DSS). The comparable validation results in the three datasets suggest the robustness of our prediction model.

Our study also has a number of limitations. First, the proposed method focuses only on the monotherapy setting, while the practical treatments of AML are usually compound combinations, e.g., the addition of FLT3 inhibitors (gilteritinib, sorafenib, quizartinib, and others) to the intensive chemotherapy or the combination of IDH inhibitors (ivosidenib and enasidenib) and venetoclax^7^. Second, the current approach of the prediction model does not allow easily further investigation into the molecular mechanism of drugs. However, in MDREAM we also introduce the characterization of the relationship between drug and molecular subtypes, which might suggest the molecular mechanism of a drug in a responsive patient. Third, the two external cohorts have a limited number of samples, but we hope we can increase the sample size in the future. Finally, BeatAML, Clinseq, and LeeAML cohorts only share a small proportion of drugs, which leads to the lack of validation of many important drugs. Over time we expect to overcome this weakness as we validate MDREAM in future patients.

The computational model of this study is based on the support vector machine (SVM) algorithm^28^ and the stacking technique^29^. Our future direction is applying deep learning algorithms. In recent years, deep learning has shown many potential advantages in drug discovery and drug response prediction^5,30,31^. A notable advantage is that deep learning can automatically extract meaningful features or can integrate prior knowledge to improve overall performance. For example, transfer learning utilizes prior knowledge from pre-trained models to improve the performance of prediction models in new datasets^32^. Mourragui et al. propose an approach to transfer drug response predictors on cell lines and patient-derived xenografts to human tumors^33^. Another potential direction is extending MDREAM for drug synergy. Some recent studies show the effectiveness of drug combinations compared to monotherapy in hypertension^34,35^. In AML, Jafari and colleagues recently developed bipartite network models to design combination therapies^36^.

## Methods

### Prediction model and genomic features for drug response of AML

The ensemble-based prediction model of MDREAM is built based on the stacking approach^29^. It consists of two layers: one for the base models ${M}_{{\mathrm{base}}}^{{D}_{i}}$ and another for the ensemble models ${M}_{{\mathrm{ensemble}}}^{{D}_{i}}$, where *D*_*i*_ is one of 122 drugs from BeatAML; see Fig. 1b. This approach can help one drug getting extra information from other drugs to improve the robustness of the prediction by utilizing the correlation of drug responses between drugs, for example, those share similar set of gene targets in the second layer. A base model ${M}_{{\mathrm{base}}}^{{D}_{i}}$ of drug *D*_*i*_ inputs genomic features from gene expression and mutation profiles into a machine-learning method to build a prediction model for the drug. The ensemble model ${M}_{{\mathrm{ensemble}}}^{{D}_{i}}$ for drug *D*_*i*_ collects the prediction results from all of the base models across drugs to build another prediction model for drug *D*_*i*_. The outputs of the ensemble models are the final prediction results of MDREAM.

The genomic features to train the base models are selected using gene expression and mutation profiles. First, the gene expression of each sample is normalized by median centering and unit variance (median = 0, sd = 1). We then focus on AML-context-driven genes, including

- AML subtype-specific genes: we utilize the findings from Mou et al.^14^, which provides a list of genes specific to 11 AML molecular subtypes. For each molecular subtype, we keep 15 top genes.
- Pathway activation score (PAS): The previous study^37^ introduces PAS to present a tumor-specific pathway activity level that is relevant to a specific drug and shows an association between PAS and drug sensitivity in AML. We calculate the PAS using the gene expression of the somatic mutations for the prediction model.
- Drug-target genes: Primary target genes of each drug are collected from DrugBank database^15^. Furthermore, we include genes from upstream and downstream of the target genes from regulatory network databases^38–41^. Theoretically, the response of a drug may be associated with gene expression of these genes.
- Mutated genes: driver mutations are targeted by multiple inhibitors; therefore, we also collect the mutations of patients to the prediction model. Gene expression of the mutated genes is used as the input for the model.
- Other AML-relevant genes: we further collect genes relevant to AML from previous studies^2,42^, FTL3 signatures^43^, and TP53 signatures^44^.

We also consider data-driven genes, which are selected based on the information from the training data, including

- Highly varying genes: 100 genes with the highest variance across the training patients.
- Correlated genes: 100 genes most correlated with the drug response.

We filter out genes which are highly correlated with other genes (*r* > 0.90 Spearman correlation) to get the final features set.

As the predictor, we use the support vector machine (SVM)^28^ with the radial kernel using the e1071 package, R version 3.6.3. The SVM is fast and flexible, but other machine-learning methods, such as the random forest^45^ or elastic net^46^ can be used. No parameter tuning for the SVM is used. The default parameter setting of SVM in the e1071 R-package is selected to work for most datasets in general. During the challenge, we tried out multiple prediction models such as random forests, elastic net, etc., as well as parameter tuning for those models. We discovered that for MDREAM, tuning the SVM parameters did not make a significant difference in overall performance but required a huge computational resource. We found that the performance mainly depends on choosing meaningful biological features rather than on tuning the parameters and selecting the models. We checked and found that the overall prediction results did not depend very much on the machine-learning method and parameter tuning (which requires a huge computational resource), but more on the set of meaningful biological features (data not shown). In the web application, we also use a similar geneset from the MDREAM model and the SVM to build the subtype prediction model for new samples.

### Confidence score for drug-response prediction

To compute the confidence score (CS) of a drug response prediction, we first define “good response” based on a threshold of drug sensitivity. In the BeatAML cohort, IC_50_≤*T*_IC50_ or ${{{\rm{AUC}}}}\le {T}_{{\mathrm{AUC}}}^{D}$ are defined as good response, where *T*_IC50_ and ${T}_{{\mathrm{AUC}}}^{D}$ are the thresholds for drug D. We select *T*_IC50_ = 1 as the threshold for IC_50_ prediction, but this can be adjusted in the MDREAM application. For the prediction of AUC, the threshold ${T}_{{\mathrm{AUC}}}^{D}$ of drug *D* is estimated using a non-parametric regression model fitting the IC_50_ and AUC values of the drug. Figure 3a illustrates the relationship between IC_50_ (x-axis) and AUC (y-axis) of *D* = dovitinib in the BeatAML cohort, which is used to estimate the ${T}_{{\mathrm{AUC}}}^{{\mathrm{Dovitinib}}}$. The fitted model (red curve) shows that ${T}_{{\mathrm{AUC}}}^{{\mathrm{Dovitinib}}}=176$ corresponds to *T*_IC50_ = 1.

Given *Pr* a predicted AUC of drug *D* for patient *P*, CS is the proportion of good-response prediction for (drug *D*, patient *P*) across bootstrap replicates of the training data. Specifically, we bootstrap the training set *N* times to build *N* bootstrap prediction models *M*_*i*_, where *i* = (1,..,*N*). Then, *N* predictions {*Pr*_1_, *Pr*_2_, ..., *Pr*_*N*_} of drug *D* for patient *P* from the bootstrap models are collected. CS is computed by

1 $${{{\rm{CS}}}}({\mathrm{Pr}})=\frac{\#\{P{r}_{i}\le {T}_{{\mathrm{AUC}}}^{D}\}}{N}.$$

The calculation of CS for IC_50_ is similar but use ${T}_{{\mathrm{IC50}}}^{D}$ for the drug sensitivity threshold. In the current study, we use *N* = 100 bootstrap replications.

### AML molecular subtypes in relation to drug response

The AML molecular subtypes are somewhat well-known among AML oncologists. So its connection to a predicted drug response is useful information to suggest possible biological reasons for the effectiveness of the drug. In this study, we focus on 11 AML distinct molecular subtypes from the recent study of Papaemmanuil et al.^2^. In the web application of MDREAM, the molecular subtype of a patient is either provided in advance by the user or predicted by a prediction model as described in the section “Prediction model and genomic features for drug response of AML”.

In the analysis of the relationship between AML molecular subtypes and drug response, we use the network enrichment analysis (NEA)^16^. NEA assesses the functional network connectivity between two genesets. It extends the traditional geneset enrichment analyses (GSEA) with informative topological information in terms of gene interaction networks. This analysis utilizes a comprehensive network containing ~1.4 million functional interactions between 16,299 distinct HUPO genes. In this context, we measure the functional connectivity between subtype-specific genes and drug-target genes.

For the first geneset, we utilize the top 25 genes of the 11 molecular subtypes reported by Mou and colleagues^14^. For the second geneset, we obtain a list of drug-target genes derived from the Genomics of Drug Sensitivity in Cancer (GDSC) cohort^20^ and DrugBank database^15^. And then, NEA simplifies the assessment of the association by defining an enrichment score as:

2 $$z=\frac{{d}_{AF}-{\overline{d}}_{AF}}{{\sigma }_{AF}}$$

where *d*_*AF*_ is the number of links between the two genesets; ${\overline{d}}_{AF}$ and *σ*_*AF*_ are the mean and standard deviation of *d*_*AF*_ respectively, which are estimated on a randomized network under the null hypothesis of no drug-subtype interaction. Then, we investigate the correlation between *z*-score of drug-subtype interaction and drug sensitivity AUC.

### Datasets

#### BeatAML cohort

The BeatAML project^11^ provides both omics data of AML, including gene expression and mutation profile, as well as drug-response assay data, from 461 patients. Gene expressions (count-per-million, CPM) of 26,086 hg19 genes and mutations of 302 genes are available. The gene expression data are normalized centrally by the median, and we keep only mutations which are recurrent in at least two samples. The drug sensitivity data consist of 47,650 records from 528 AML patients across 122 compounds. A total of 32,263 records from 337 patients with RNA-Seq data are used for this study. Both measurement metrics of drug response, including IC_50_ and AUC are included.

For evaluating the prediction model, we split data into a training set (*n* = 278) and a testing set (*n* = 183) according to the center ID of the samples, such that no center IDs overlap between the two sets. The samples of each set are listed in Supplementary Data 3. Further evaluation is implemented using 10-fold cross-validation on the training set.

#### Clinseq cohort

This is a Swedish AML cohort^47^, which comprises 315 AML patients from 1997 to 2014. The RNA-seq samples were sequenced by Illumina HiSeq 2500 platform and gene expressions of 20,000 genes were estimated by XAEM^48^ using hg19 annotation. The drug data contains the drug sensitivity of 528 drugs on 45 patients measured by drug sensitivity score (DSS)^12^. A total of 76 drugs shared between Clinseq and BeatAML is included in the analysis.

#### LeeAML cohort

This cohort is based on a pilot clinical trial on relapsed or refractory acute leukemia patients in the United States from 2015 to 2021, which included drug sensitivity profiles and gene expressions of 54 patients^49^. Our study uses only the published gene expressions and drug data from 12 patients^10^. Two RNA-seq replicates for each patient were prepared using Illumina TruSeq stranded mRNA kit genes. The final gene expression (in fragments per kilobase of transcript per million mapped fragments, FPKM) of 20,060 genes for each patient was averaged from Cufflink’s output of two replicates. The drug data includes 1872 drug sensitivity (AUC) profiles of 156 drugs.

### Reporting summary

Further information on research design is available in the Nature Research Reporting Summary linked to this article.

## Supplementary information

Supplementary Figure

REPORTING SUMMARY

Supplementary Data

## Data Availability

The public data were available from their original studies: Gene Expression Omnibus (GEO) under accession numbers GSE108003 for the LeeAML dataset^10^, dbGaP accession ID phs001657.v1.p1 for the BeatAML dataset^11^. The demographic information and somatic mutations of the Clinseq dataset^47^ are publicly available in the Clinseq AML repository at Zenodo 10.5281/zenodo.292986. The drug data from the in-house Clinseq dataset^47^, used here for model validation, is not publicly available at present, as it is part of an ongoing main study that generates the data. For further details about the Clinseq drug data, please contact the corresponding author.

## Code availability

The web-based implementation of MDREAM is publicly available at https://www.meb.ki.se/shiny/truvu/MDREAM/. The pipeline for training MDREAM is available at https://www.github.com/tracquangthinh/MDREAM.
