# Supplementary material for: Prediction model for drug response of acute myeloid leukemia patients
Source: NPJ Precis Oncol. 2023 Mar 24;7:32. doi: 10.1038/s41698-023-00374-z (PMC10039068; doi:10.1038/s41698-023-00374-z)
Supplement: Supplementary file 1 — Supplementary Figure [file 41698_2023_374_MOESM1_ESM.pdf]

Supplementary Figures for

Prediction model for drug response of acute myeloid leukemia patients

Quang Thinh Trac, Yudi Pawitan, Tian Mou, Tom Erkers, Päivi Östling, Anna Bohlin, Albin Österroos, Mattias Vesterlund, Rozbeh Jafari, Ioannis Siavelis, Helena Bäckvall, Santeri Kiviluoto, Lukas M. Orre, Mattias Rantalainen, Janne Lehtiö, Sören Lehmann, Olli Kallioniemi, and Trung Nghia Vu

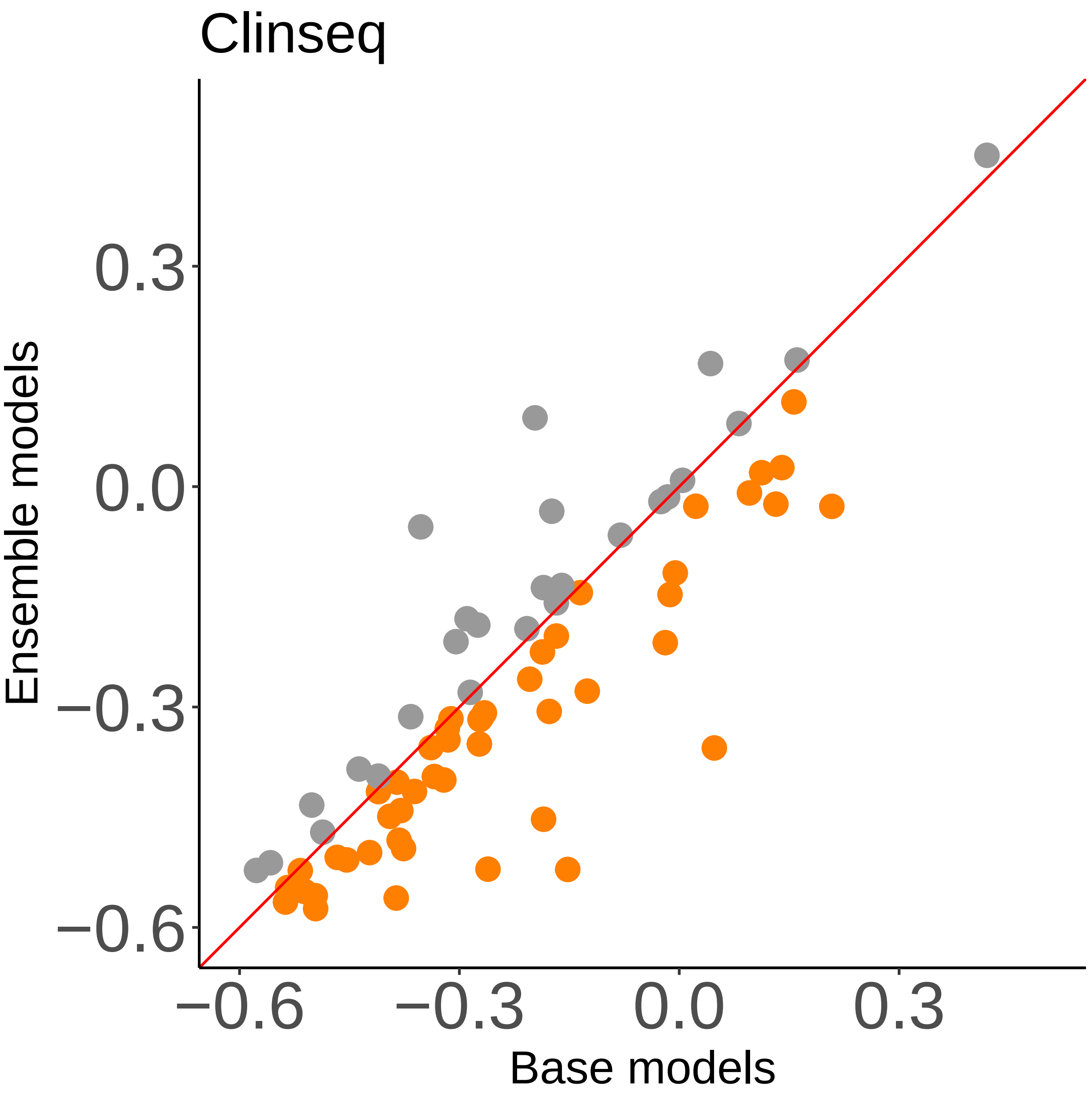

Supplementary Figure 1: The comparison of prediction performance between the base models (x-axis) and the ensemble models (y-axis) in the Clinseq cohort using the correlation between predicted values and observed values of individual drugs. Here, orange points below the diagonal line indicates better correlation archived by the ensemble models since the AUC and drug sensitivity score (DSS) have opposite directions.

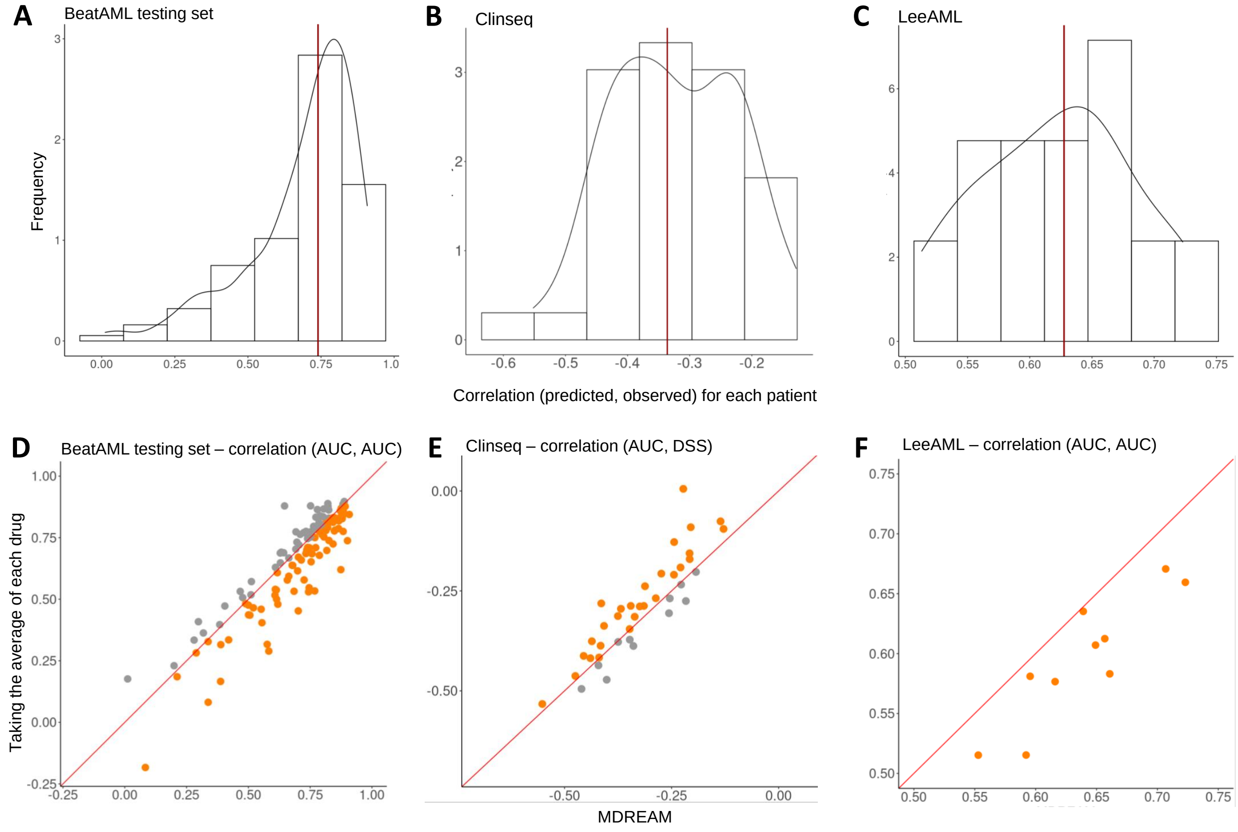

Supplementary Figure 2: **ABC** - The distribution of the correlation between predicted and observed response for all individual patients in the BeatAML testing (A), Clinseq (B) and LeeAML (C) datasets. **DEF** - Comparison of performance between MDREAM (x-axis) and the drug-average model (y-axis) in the BeatAML testing set (D), Clinseq (E) and LeeAML (F). Each point represents the correlation between the predicted and observed values of an individual patient.

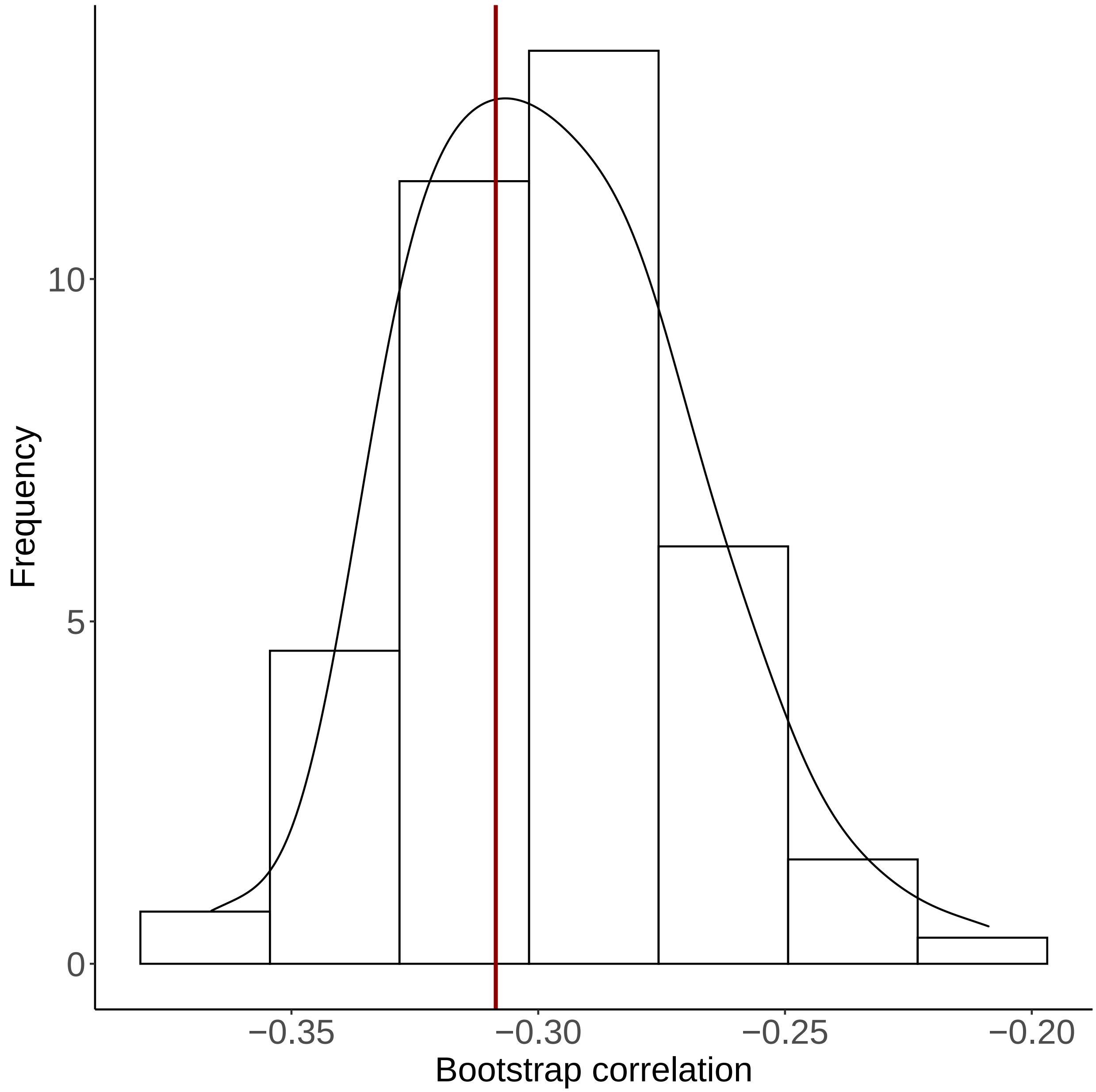

Supplementary Figure 3: The distribution of the bootstrap population of the median correlation between the predicted AUC and the observed DSS across drugs in the Clinseq data. The red line describe the median correlation across drugs in Clinseq.

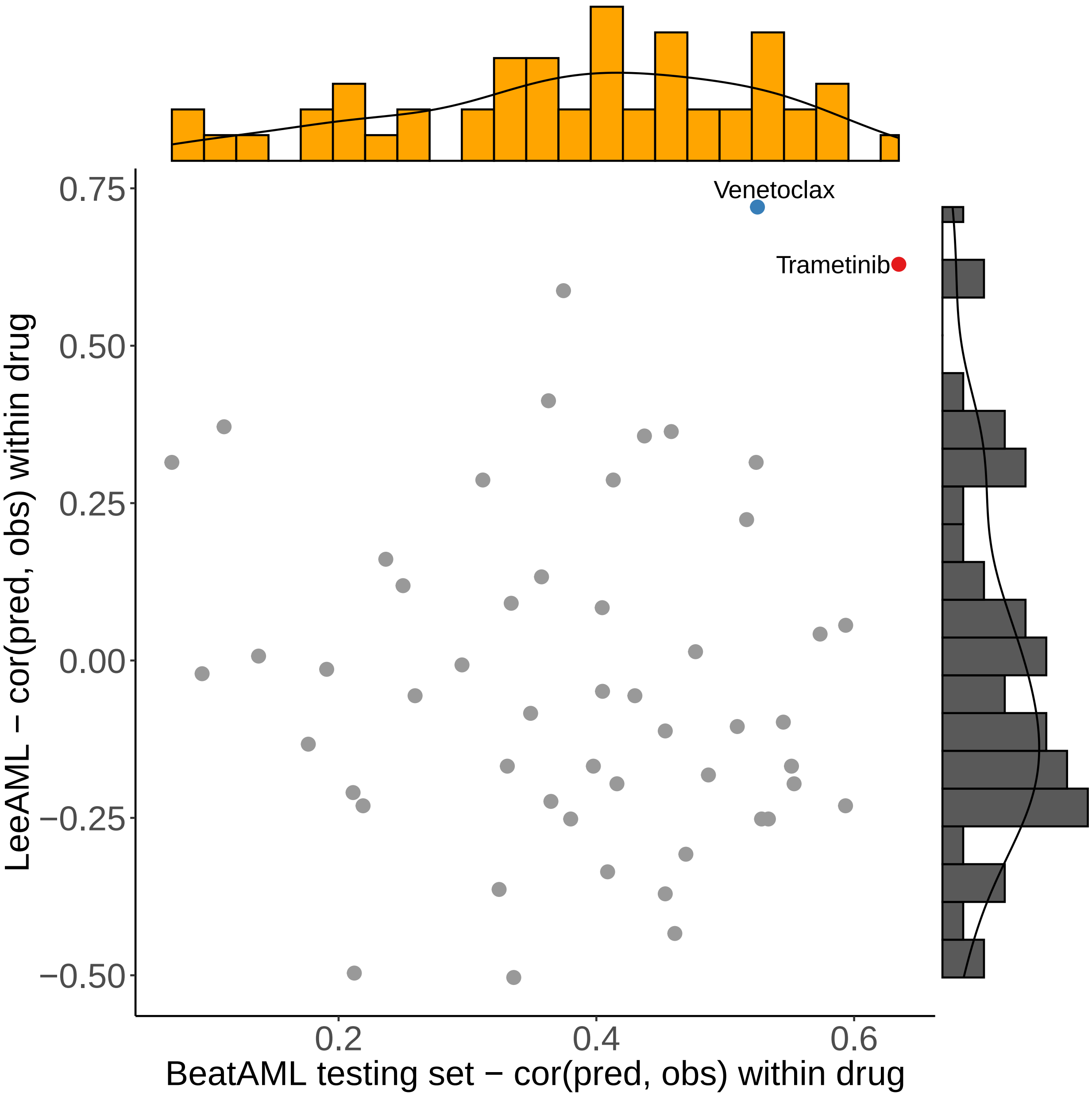

Supplementary Figure 4: Prediction performances of the BeatAML testing set (x-axis) versus the LeeAML dataset (y-axis) across 52 overlapping drugs using the correlation between predicted values and observed values. The drugs at the top right indicate the drugs with good predictions in both cohorts.

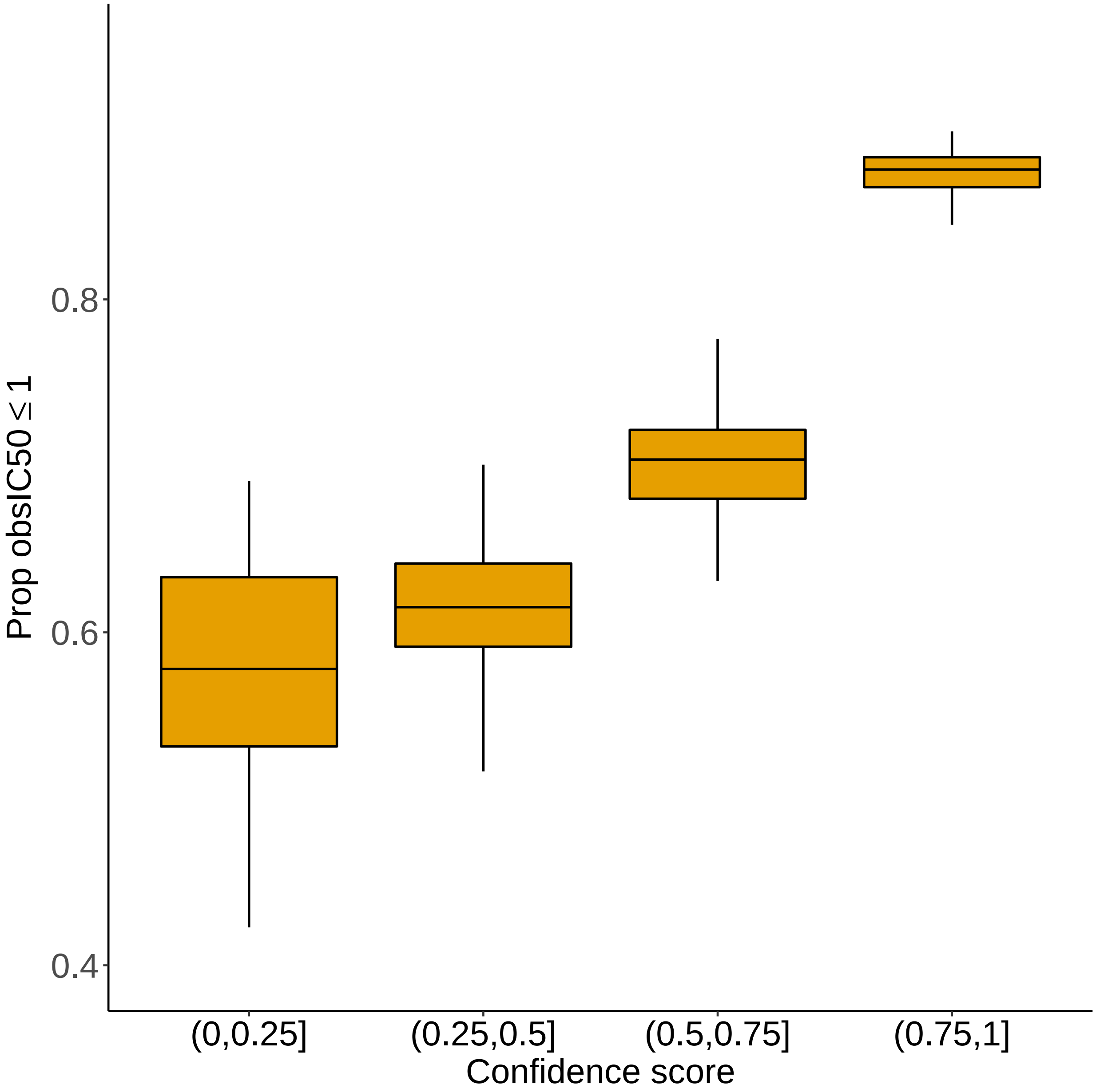

Supplementary Figure 5: Boxplots show the correlation between confidence score and proportion of good-response predictions in the BeatAML testing set. Each box plot displays the interquartile range (IQR) between the 25th percentile (the lower boundary) and the 75th percentile (the upper boundary). The center line of the box presents the median, and the whiskers are within the 1.5 IQR value. The x-axis presents 4 quartiles of the confidence scores and the y-axis shows the proportion of good-response by IC50 of drugs

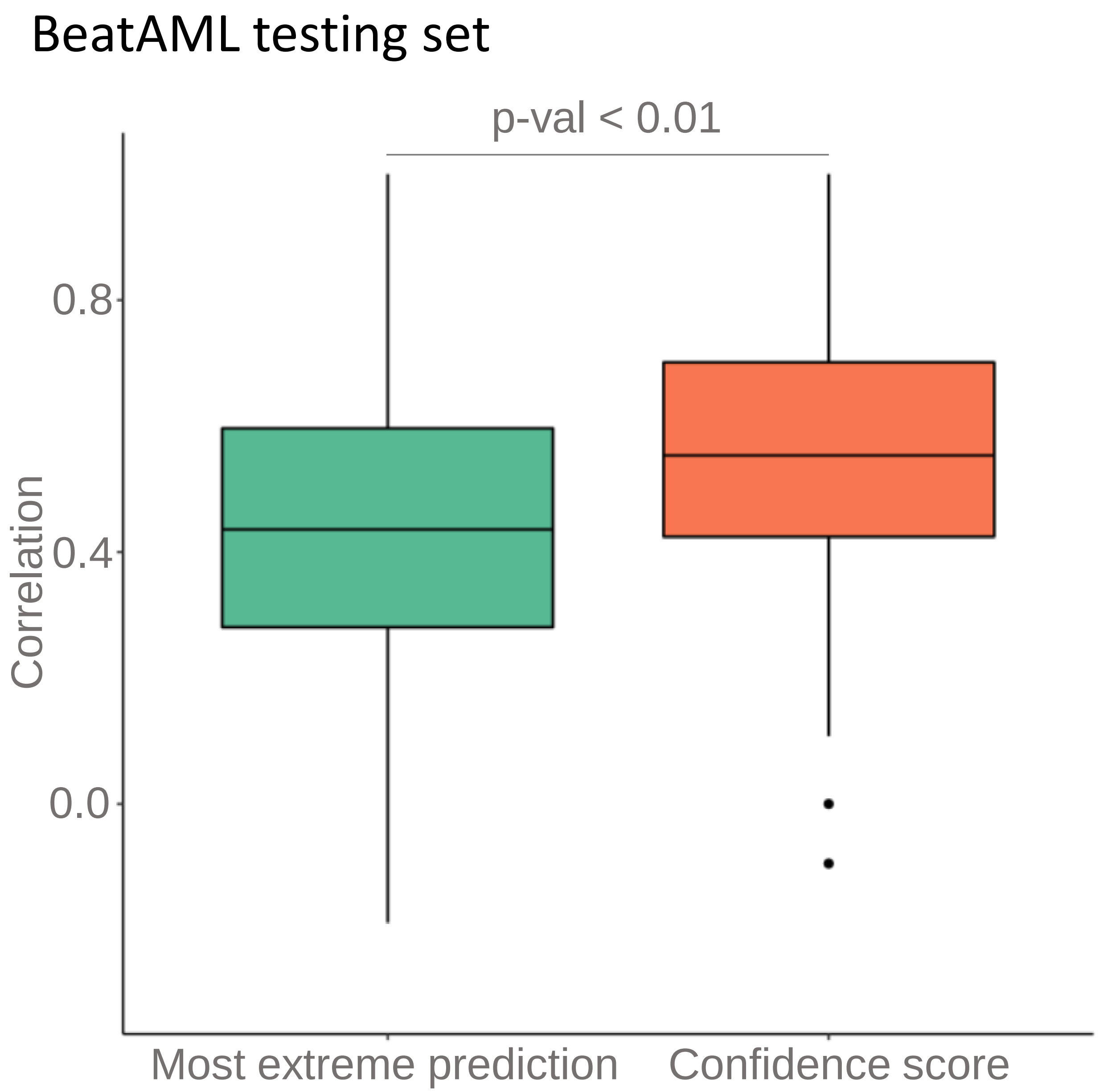

Supplementary Figure 6: Boxplots show the comparison between confidence score and most extreme predictions. Each box plot displays the interquartile range (IQR) between the 25th percentile (the lower boundary) and the 75th percentile (the upper boundary). The center line of the box presents the median, and the whiskers are within the 1.5 IQR value. The boxplots include the correlation between predicted and observed AUCs of patients in the BeatAML testing set. For each patient, K drugs with confidence score > 0.75 are collected for the confidence score group and top K drugs ranked by the predicted AUCs are used for the group of most extreme predictions.

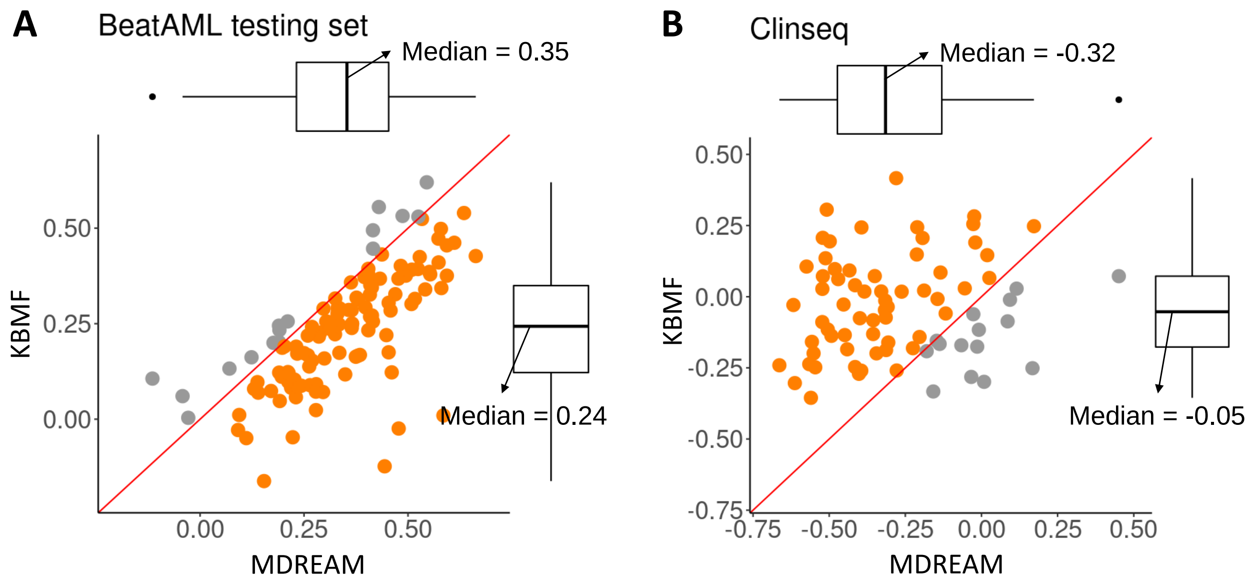

Supplementary Figure 7: Comparison of performance between MDREAM (x-axis) and Kernelized Bayesian Matrix Factorization - KBMF (y-axis) in the BeatAML testing set (A) and the Clinseq dataset (B). Each point presents the correlation between the predicted (AUC) and observed (AUC for BeatAML and DSS for Clinseq) values of a drug. Boxplots present the summary of these correlations. Each box plot displays the interquartile range (IQR) between the 25th percentile (the lower boundary) and the 75th percentile (the upper boundary). The center line of the box presents the median, and the whiskers are within the 1.5 IQR value. The orange points indicate the drugs with better performance for MDREAM.

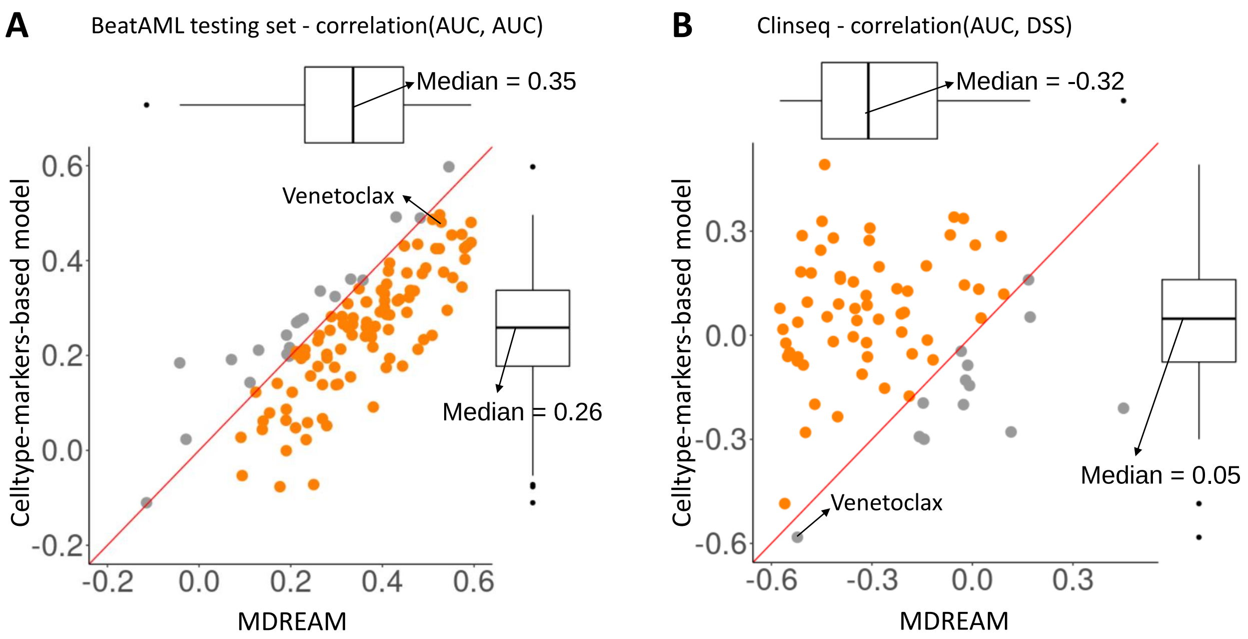

Supplementary Figure 8: Performance comparison between MDREAM (x-axis) and the celltype-markers-based model (y-axis) using the BeatAML testing set (A) and the Clinseq dataset (B). Each point presents the correlation between the predicted (AUC) and observed (AUC for BeatAML and DSS for Clinseq) values of a drug. Boxplots present the summary of these correlations. Each box plot displays the interquartile range (IQR) between the 25th percentile (the lower boundary) and the 75th percentile (the upper boundary). The center line of the box presents the median, and the whiskers are within the 1.5 IQR value. The orange points indicate the drugs with a better performance for MDREAM.

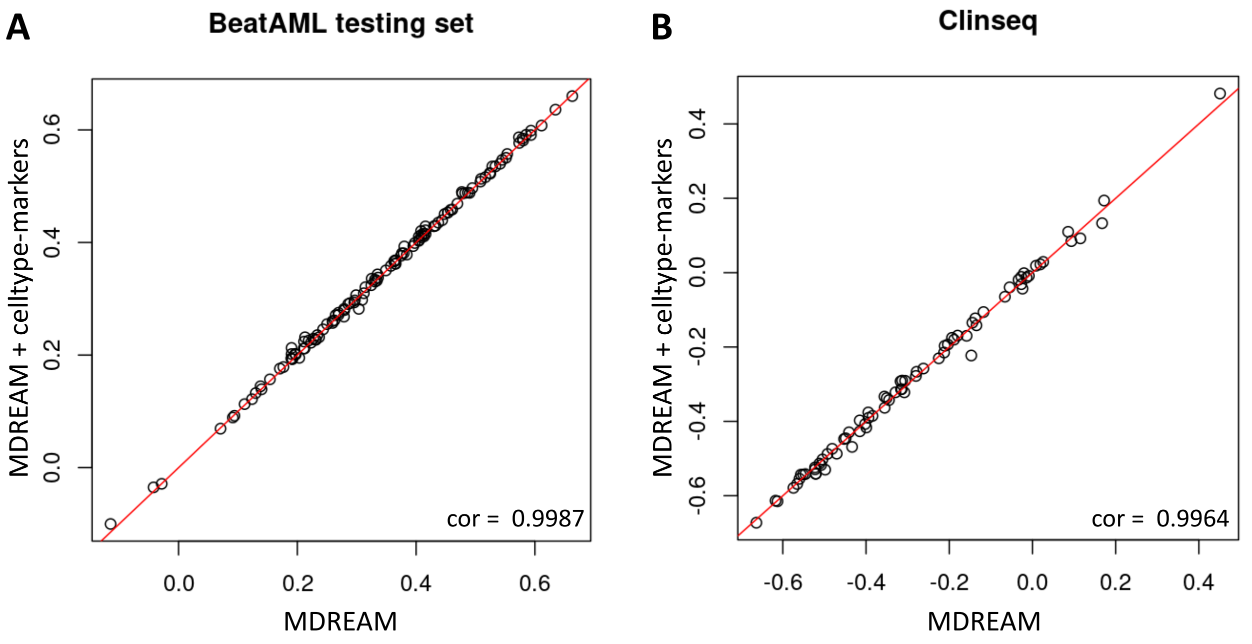

Supplementary Figure 9: Comparison of performance between the original MDREAM model (x-axis) and the MDREAM model including celtype gene markers (y-axis) in the BeatAML testing set (A) and the Clinseq dataset (B). Each point shows the correlation between the predicted and observed values of an individual drug.

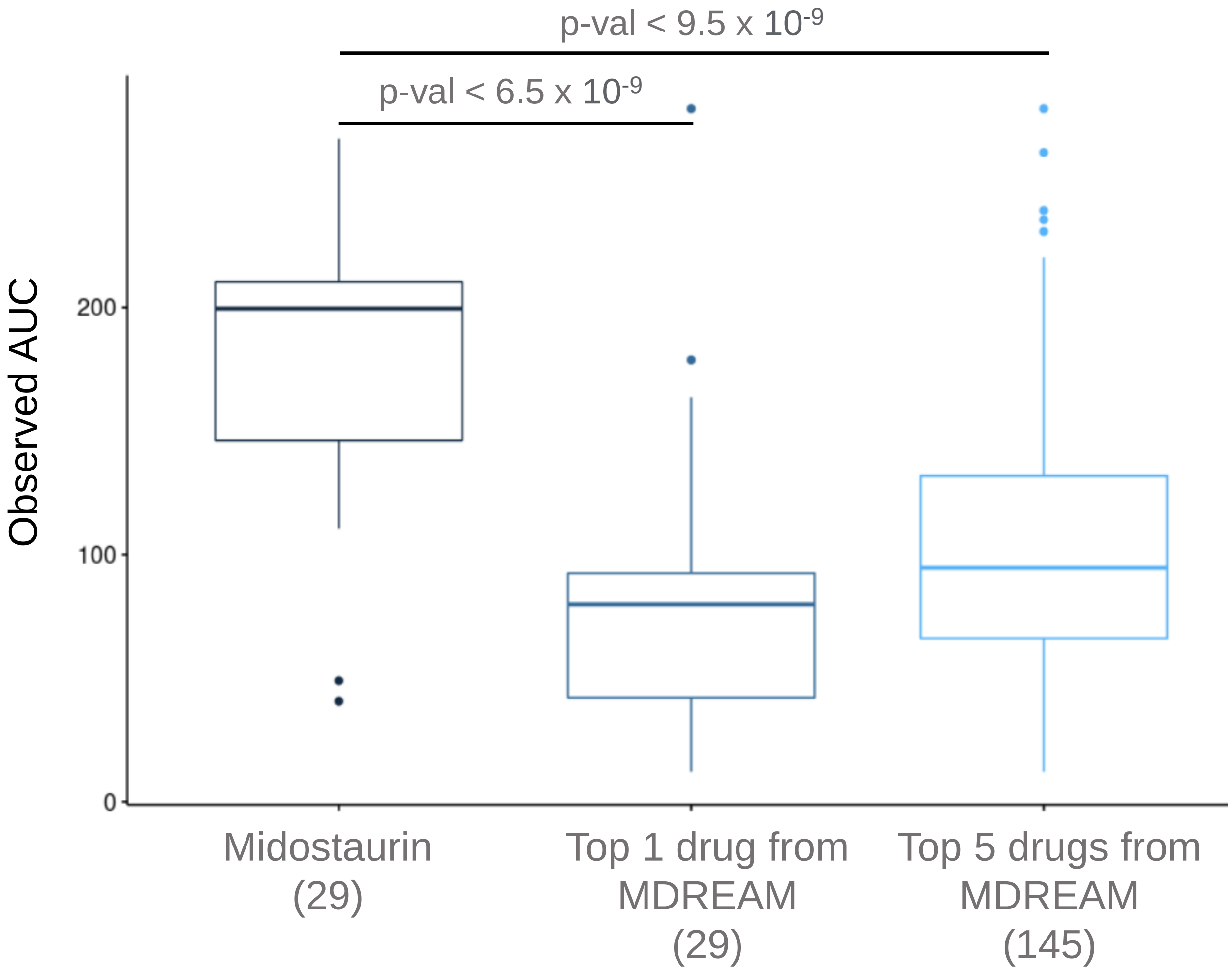

Supplementary Figure 10: Boxplots show the comparison of observed drug responses on 29 patients with FLT3 treated by Midostaurin, top-1 drug, and top-5 drugs with the most confidence scores identified by MDREAM. Each box plot displays the interquartile range (IQR) between the 25th percentile (the lower boundary) and the 75th percentile (the upper boundary). The center line of the box presents the median, and the whiskers are within the 1.5 IQR value.
